# Differences in Anxiety-Like Behavior within a Batch of Wistar Rats Are Associated with Differences in Serotonergic Transmission, Enhanced by Acute SRI Administration, and Abolished By Serotonin Depletion

**DOI:** 10.1093/ijnp/pyv018

**Published:** 2015-04-15

**Authors:** Jakob Näslund, Erik Studer, Robert Pettersson, Melker Hagsäter, Staffan Nilsson, Hans Nissbrandt, Elias Eriksson

**Affiliations:** Department of Pharmacology, Institute of Neuroscience and Physiology at the Sahlgrenska Academy, University of Gothenburg, Gothenburg, Sweden (Dr Näslund, Mr Studer, Mr Pettersson, Drs Hagsäter, Nissbrandt, and Eriksson); Institute of Mathematical Sciences, Chalmers University of Technology, Gothenburg, Sweden (Dr Nilsson).

**Keywords:** Serotonin, anxiety, serotonin reuptake inhibitors, tryptophan hydroxylase 2, elevated plus maze

## Abstract

**Background:**

The anxiety-reducing effect of long-term administration of serotonin reuptake inhibitors is usually seen only in subjects with anxiety disorders, and such patients are also abnormally inclined to experience a paradoxical anxiety-enhancing effect of acute serotonin reuptake inhibition. These unique responses to serotonin reuptake inhibitors in anxiety-prone subjects suggest, as do genetic association studies, that inter-individual differences in anxiety may be associated with differences in serotonergic transmission.

**Methods:**

The one-third of the animals within a batch of Wistar rats most inclined to spend time on open arms in the elevated plus maze were compared with the one-third most inclined to avoid them with respect to indices of brain serotonergic transmission and how their behavior was influenced by serotonin-modulating drugs.

**Results:**

“Anxious” rats displayed higher expression of the tryptophan hydroxylase-2 gene and higher levels of the tryptophan hydroxylase-2 protein in raphe and also higher levels of serotonin in amygdala. Supporting these differences to be important for the behavioral differences, serotonin depletion obtained by the tryptophan hydroxylase-2 inhibitor *p*-chlorophenylalanine eliminated them by reducing anxiety in “anxious” but not “non-anxious” rats. Acute administration of a serotonin reuptake inhibitor, paroxetine, exerted an anxiety-enhancing effect in “anxious” but not “non-anxious” rats, which was eliminated by long-term pretreatment with another serotonin reuptake inhibitor, escitalopram.

**Conclusions:**

Differences in an anxiogenic impact of serotonin, which is enhanced by acute serotonin reuptake inhibitor administration, may contribute to differences in anxiety-like behavior amongst Wistar rats.

## Introduction

Many authors have suggested inter-individual differences in serotonergic transmission to be the cause of inter-individual differences in proneness for anxiety (Lesch et al., 1996; Maron et al., 2012; Mosienko et al., 2012; Araragi and Lesch, 2013). The specific response to serotonin reuptake inhibitors (SRIs) displayed by subjects with anxiety disorders may be regarded as indirect support for this assumption. Thus, while traditional anxiolytic drugs acting by modulation of the GABA A receptor complex, such as barbiturates and benzodiazapines, exert nonspecific sedation also in nonanxious subjects and also dampen rational fear, long-term administration of SRIs results in an impressive symptom reduction in patients with anxiety disorders (Den Boer and Westenberg, 1988; Modigh et al., 1992; Nutt et al., 1999; Zohar and Westenberg, 2000) while exerting no (Furlan et al., 2004) or merely subtle fear- and anxiety-reducing effects in healthy controls (Simmons and Allen, 2011). Reciprocally, while acute administration of SRIs (Ramos et al., 1993; Nutt et al., 1999; Sinclair et al., 2009) or the serotonin releasing agent fenfluramine (Targum and Marshall, 1989) often increases anxiety in patients with anxiety disorders, such as panic disorder, and in subjects with anxiety-related personality traits (Rammsayer and Netter, 1990), this response is generally absent or mild in nonanxious subjects exposed to these drugs.

In the same vein, genetic studies have revealed serotonin-related genes to be associated with anxiety-related personality traits (Lesch et al., 1996; Melke et al., 2003; Sen et al., 2004) and anxiety-related endophenotypes such as amygdalar reactivity (Hariri et al., 2002; Furmark et al., 2009). Methodological limitations, however, still hamper the possibility to measure the status of brain serotonergic neurotransmission in humans, and there is hence limited support for aberrations in serotonergic activity in anxiety-prone subjects and also no consensus regarding if serotonin should be regarded mainly as anxiety enhancing (Eison, 1990; Graeff and Zangrossi, 2010; Andrade et al., 2013) or anxiety reducing (Nutt et al., 1999; Graeff and Zangrossi, 2010; Donner et al., 2012), or if it may even exert both effects (Graeff and Zangrossi, 2010). To what extent long-term administration of SRIs enhances (Nutt et al., 1999; Bell et al., 2002) or dampens (Salchner and Singewald, 2006) a serotonergic influence on anxiety-regulating circuits, or may exert both effects (Graeff and Zangrossi, 2010), likewise remains a matter of controversy.

Numerous animal studies suggest that manipulation of brain serotonergic neurotransmission, obtained by drugs (Pinheiro et al., 2007) or genetic manipulation (Fernandez and Gaspar, 2012; Mosienko et al., 2012; Araragi and Lesch, 2013), leads to changes in anxiety-related behavior as reflected, for example, by avoidance of open arms in the elevated plus maze (EPM). However, to what extent inter-individual differences with respect to EPM behavior within a batch of experimental rats is associated with inter-individual differences in brain serotonergic transmission, and if eliminating the influence of serotonin may abolish such behavioural differences, is unknown.

Previous studies of EPM performance in outbred Wistar rats support the existence of relatively stable (test vs retest) inter-individual variations that are sufficiently robust to justify the characterization of the rats as more or less “anxious” (Schneider et al., 2011). These differences are associated with other aspects of behavior (Ho et al., 2002; Borta et al., 2006) and brain neurochemistry (Schwarting et al., 1998), and they have also provided the basis for the breeding of strains characterized by low or high anxiety-like behavior (Liebsch et al., 1998). The aim of the present study was to utilize these inter-individual differences in Wistar rats with respect to EPM behavior to shed further light on 3 long-debated issues: 1) are inter-individual differences in anxiety to some extent caused by inter-individual differences in serotonergic transmission, 2) is the possible contribution of serotonergic neurotransmission to inter-individual differences in anxiety best described as anxiety *promoting* or anxiety *reducing*, and 3) is the influence of SRIs, administered acutely or on a continuous basis, respectively, best described as a *facilitation* or a *dampening* of a serotonergic influence on anxiety-generating circuits.

## Materials and Methods

### Animals

Male Wistar rats (Taconic, Ejby, Denmark), aged 10 to 11 weeks at arrival, were housed with a 12-h-light/-dark cycle (lights on at 6 am) and with standard chow and water available ad libitum. The animals were allowed 1 week of acclimatization after arrival before being subjected to any behavioral tests. In all experiments, animals were subdivided on the basis of how they performed in the EPM, the one-third most prone to spend time on the open arm constituting one group, presumably characterized by low anxiety (high open arm, HO) and the one-third most prone to avoid it constituting the other, tentatively more anxious group (low open arm, LO). The middle group was thus excluded from all further analyses in order to avoid misclassification of animals belonging to this group but being close to one of the extreme groups.

All procedures were carried out with approval of the local ethics committee and in accordance with institutional guidelines.

### Experimental Outline

#### Experiment I

Forty-eight animals were tested in the EPM for categorization into HO (one-third) and LO (one-third) rats 2 weeks before being subjected to a test of unconditioned noise burst-elicited startle (lasting for 15 minutes and comprising 30 bursts with 30-second intervals at 95 dB and with 20ms duration per burst; Med Associates, St. Albans, VT) followed 1 week later by a forced-swim test (comprising two 10-minute sessions separated by 24 hours). One week after the forced-swim test, the animals were killed and their brains extracted for assessment of gene expression using real-time PCR (TaqMan), tryptophan hydroxylase 2 (TPH2) levels using Western blot, and serotonin and 5-hydroxyindoeacetic acid (5-HIAA) levels using high-pressure liquid chromatography (HPLC) (see supplementary Materials and Methods).

#### Experiment II

Sixty animals were pretested in the EPM for categorization into HO (one-third) and LO (one-third) rats. After 3 weeks, treatment with the tryptophan hydroxylase inhibitor para-chlorophenylalanine (p-CPA) was initiated. Animals were again tested in the EPM 24 hours after the last injection. The following day, the animals were sacrificed; the brains from one-third of them were extracted for validation of serotonin depletion.

#### Experiment III

One hundred twenty animals were pretested in the EPM for categorization into HO (one-third) and LO (one-third), whereupon one-half of the animals in each group received escitalopram p.o. in food pellets and the other one-half control pellets. After 5 weeks of treatment, the animals received one injection of paroxetine or vehicle 1 hour before a second EPM session was undertaken.

### Drugs

p-CPA (Sigma-Aldrich, St Louis, MO) was dissolved in 0.9% saline and administered i.p. as one injection of 300mg/kg per day for 3 days with the last injection being given 24 hours before the EPM test. Escitalopram oxalate (Shodana Labs, Hyderabad, India) was admixed into food pellets (Lantmännen, Kimstad, Sweden) at a concentration (0.65g/kg) aimed at providing a daily dose of 25 to 30mg/kg (El Khoury et al., 2006). Paroxetine hydrochloride (Jai Radhe Chemicals, Ahmedabad, India) was dissolved in 0.9% saline and administered s.c. at a dose of 10mg/kg 1 hour before the EPM test.

### EPM

A standard black acrylic plastic rat EPM (Med Associates) was placed in a quiet room with a light level in the center of the maze of 35 lx. All tests lasted for 5 minutes. In the experiments comprising 2 tests, the EPM apparatus was placed in a room new to the animals at session 2 in order to minimize habituation effects (Schneider et al., 2011).

### Biochemical Analyses

See supplementary Materials and Methods.

### Statistical Analyses

Student’s *t* test was used to compare groups with respect to biochemical data. Due to non-normal distribution of the relevant behavioral parameters in drug-treated animals, EPM data were log-transformed according to the formula log10(x+1), with x being the behavioral parameter in question, before being analyzed using ANOVA followed by LSD posthoc test. Interactions were tested using 2-way ANOVA when appropriate. Pearson correlation was used to calculate correlations.

## Results

### Test-Retest Correlations

Justifying the categorization of rats into more or less anxious, we could, by examining the test-retest stability with respect to EPM performance in animals that had obtained no active pharmacological treatment between the tests, confirm the previous observation (Schneider et al., 2011) of relatively stable inter-individual differences within an outbred batch of Wistar rats (experiment II, n=20, r=0.63, *P*=.003; experiment III, n=20, r=0.68, *P*=.001). In line with this, 18 of 20 (experiment II) and 16 of 20 (experiment III) animals in the nontreated groups were found to be in the same HO/LO group in the second EPM session as in the first.

### Relationship to Other Behavioral Tests

Experiment I revealed that EPM behavior (time spent on open arms) does not correlate with average startle amplitude (n=48, r=0.02, *P*=.9) or with immobility in the forced-swim test (n=48, r=-0.08, *P*=.6); in the same vein, no differences between HO and LO animals with respect to startle response (average startle amplitude: HO 322.21±58.27, LO 290.56±43.8, *P=*.6, n=16 per group, data given as means ±SEM) or FST performance (seconds of immobility: HO 80.25±7.24, LO 98.0±9.67, *P=*.15, n=16 per group, data given as means ±SEM) were found.

### Biochemical Analyses

**Figure 1.**
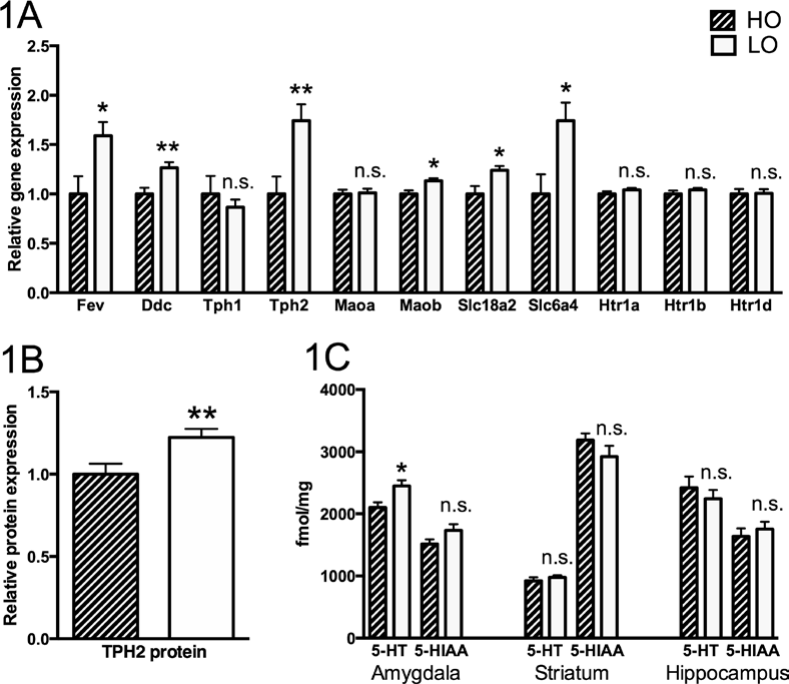
Expression of serotonin-related genes in the raphe nuclei (A), levels of the tryptophan hydroxylase 2 (TPH2) protein in the raphe nuclei (B), and levels of serotonin (5-HT) and 5-hydroxyindoeacetic acid (5-HIAA) in amygdala, hippocampus, and striatum (C) (n=10 in all groups in A and B, n=6 in all groups in C). In A and B, values of low open arm (LO) rats are expressed as geometric means ±SEM relative to the high open arm (HO) group, the mean of which is set to 1. In C, values are given as means ±SEM. Significance symbols (*) associated with a specific bar represent levels of significance for the difference between HO and LO animals: *n.s.* nonsignificant, *P<.05, **P<.01.

A comparison of LO and HO animals revealed the LO rats to be characterized by significantly higher raphe expression of several genes expressed more or less exclusively by serotonergic neurons, including the TPH2 gene (Figure 1A), but showed no difference with respect to serotonin-related genes that are also expressed by other cells. TPH2 levels being elevated in the raphe region of LO animals was confirmed using Western blot (Figure 1B). Moreover, analysis of serotonin and the serotonin metabolite 5-HIAA using HPLC revealed higher serotonin levels in the amygdala of LO rats but no difference with respect to 5-HIAA levels and no differences in hippocampus and striatum (Figure 1C).

### Behavioral and Biochemical Effects of p-CPA

**Figure 2.**
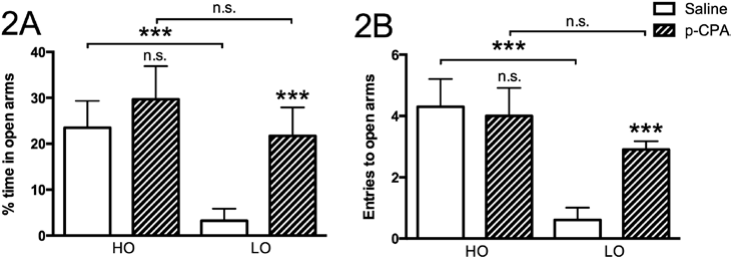
Percentage of time spent on open arms (A) and number of entries made onto open arms (B) by animals receiving either saline or para-chlorophenylalanine (p-CPA). Values are given as means ±SEM. n=10 in all groups. Significance symbols (*) associated with a specific bar represent level of significance for the difference between rats of the same group (high open arm [HO] and low open arm [LO], respectively) given saline or p-CPA, respectively: *n.s.* nonsignificant, **P<.01, ***P<.001.

Administration of the tryptophan hydroxylase inhibitor (p-CPA) led to an increase in time spent on open arms (Figure 2A) and entries onto open arms (Figure 2B) in LO rats but exerted no corresponding effect in the HO group, hence abolishing the behavioral difference between the 2 groups. HPLC analysis of serotonin content in brain stem and amygdala confirmed a robust decrease of both serotonin (forebrain: -95.2±0.7%; *P*≤.001; brainstem: -97.6±0.4%; *P*≤.001) and 5-HIAA (forebrain: -97.8±0.6%; brainstem: *P*≤.001; -93.0±1.1%; *P*≤.001) levels (n=9–10/group) in p-CPA–treated animals (n=9–10/group) with no tendencies for any differences between LO and HO animals in this regard.

### Behavioral Response to Acute SRI Administration

**Figure 3.**
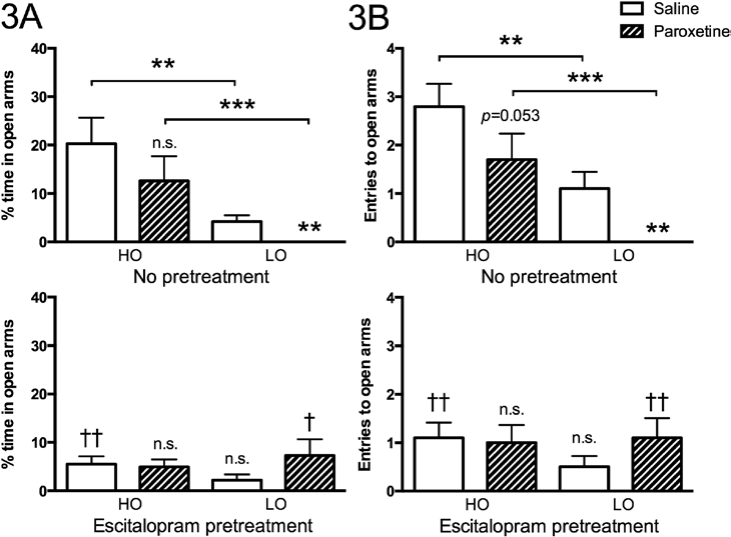
Time spent on (A) and entries made onto (B) open arms by animals receiving either saline or paroxetine. The upper graphs show otherwise untreated animals; the lower graphs show animals administered escitalopram p.o. for 5 weeks. Values are given as means ±SEM. (n=10 in all groups). Significance symbols (*/n.s.) associated with a specific bar in the upper graph represent level of significance for the difference between rats of the same group (high open arm [HO] and low open arm [LO], respectively) given saline or paroxetine. Significance symbols (†/n.s.) associated with a specific bar in the lower graphs represent differences between the indicated group (having received escitalopram) and the corresponding group in the upper graphs (having received control pellets). *n.s.* nonsignificant, *†*P*<.05, ††*P*<.01, ***P<.001. Comparisons of the different groups displayed in the lower graph revealed no significant differences.

Acute administration of paroxetine exerted an anxiogenic effect, that is, decreased the time spent on, as well as entries onto, open arms in otherwise untreated LO animals. No such effect was seen in the corresponding HO group, albeit a trend towards decreased entries to open arms was observed (Figure 3A-B, upper graphs).

### Behavioral Response to Subchronic SRI Administration

Pretreatment with escitalopram (p.o.) for 5 weeks caused a modest and nonsignificant reduction in time spent on open arms in otherwise untreated LO rats but totally prevented the anxiogenic-like effect of acute administration of another SRI, paroxetine, in these animals, the interaction between escitalopram and paroxetine being significant (*P*<.007, F=8.281, df=1). HO rats given subchronic escitalopram displayed reduced time on open arms; like otherwise untreated HO rats and escitalopram-treated LO rats, they did not, however, display an anxiogenic-like response to acute administration of paroxetine. Subchronic escitalopram administration hence eliminated all differences with respect to open arm duration and entries between LO and HO animals (Figures 3A-B, lower graphs).

### Effects of Escitalopram and Paroxetine on Entries into Closed Arms

To evaluate if the effect of acute SRI administration on anxiety as assessed using EPM could be secondary to an effect on locomotion in general, we also measured entries into closed arms. This parameter was not different in untreated HO rats (3.9±0.7) compared with untreated LO animals (4.0±0.9; n.s.). Administration of paroxetine did not cause a significant reduction in the number of entries into closed arms in either HO (3.4±0.4: n.s.) or LO rats (2.3±0.6; n.s.); hence, there were no significant differences between any of the groups displayed in Figure 3A with respect to closed arms entries. Subchronic administration of escitalopram did not influence closed arm entries in HO rats (4.7±0.6, n.s.) but exerted a modest reduction in LO rats (2.3±0.5) that was nonsignificant when these animals were compared with controls not given escitalopram but of sufficient magnitude to make this group differ significantly from the corresponding HO group (*P*<.01). Administration of paroxetine to escitalopram-treated LO rats, however, counteracted this effect of escitalopram (4.1±0.67); this group hence differed neither from HO rats given escitalopram plus NaCl nor from HO rats given escitalopram plus paroxetine, but did differ from LO rats given escitalopram plus NaCl (*P*<.05).

## Discussion

This study suggests that inter-individual differences in anxiety in a batch of male Wistar rats may be partly explained by differences in an anxiogenic influence of serotonin. While animals prone to anxiety-related behavior (LO) hence displayed indices of enhanced serotonergic transmission, including enhanced TPH2 expression, arresting serotonergic transmission by inhibiting this enzyme reduced anxiety-like behavior selectively in these rats, thereby making the differences in behavior between LO and HO animals disappear. Further, similar to the human situation and in line with the assumption that nonanxious and anxious animals differ with respect to the magnitude of an anxiogenic-like serotonergic influence, we found animals with high baseline anxiety-like behavior to display a greater anxiogenic-like response to acute administration of an indirect serotonin agonist, the SRI paroxetine. Finally, long-term administration of an SRI, which has previously been suggested to cause an adaptive downregulation of the anxiogenic influence of serotonin (Salchner and Singewald, 2006; Graeff and Zangrossi, 2010), was found to blunt the anxiogenic-like effect of acute SRI administration in LO animals, hence making them similar to HO rats in this regard. The results support the view that inter-individual differences with respect to an anxiogenic influence of serotonin are important for inter-individual differences in anxiety-related behavior and that the anxiety-reducing effect of long-term SRI administration may be attributed to a downregulation of such an influence.

Our claim that LO animals are characterized by enhanced serotonergic activity is based mainly on the observation that they display enhanced expression of the gene encoding the rate-limiting enzyme for the synthesis of serotonin, TPH2, in the region where serotonergic cell bodies are situated, the raphe nuclei, and that this increase in TPH2 expression was confirmed by means of Western-blot assessment of the TPH2 protein. Our findings are in line with previous studies showing that raphe TPH2 expression is enhanced in rodents rendered anxious by genetic modulation of nonserotonergic genes (Jahanshahi et al., 2011) or by subchronic infusion of a corticotropin-releasing agent into the basolateral amygdaloid complex (Donner et al., 2012). Moreover, the importance of nongenetic factors in this context is illustrated by the observations that chronic restraint stress, maternal deprivation during early life combined with social defeat at adulthood, and neonatal administration of lipopolysaccharides are all interventions that produce both anxiety-like behavior in the EPM and elevated TPH2 expression in rodents (Chamas et al., 1999; Gardner et al., 2009; Sidor et al., 2010). In contrast, reduced raphe TPH2 expression was reported in Wistar rats displaying a low degree of exploratory behavior; this study, however, did not employ the EPM paradigm but a test based on exploration of novel and familiar objects and assumed to reflect both motivation and anxiety (Alttoa et al., 2010).

The apparent enhancement of serotonergic transmission in animals displaying enhanced anxiety-like behavior could be regarded either as support for an anxiety-promoting role of serotonin or explained in terms of compensatory mechanisms aiming to dampen the influence of various anxiety-promoting circuits. In the present study, we show a TPH2 inhibitor, p-CPA, to reduce anxiety-like behavior in anxious LO rats but not in nonanxious HO animals, hence eliminating the behavioral difference between the 2 groups. While the anxiolytic-like effect of p-CPA in this paradigm, which supports an anxiogenic-like influence of this transmitter, is well known (Treit et al., 1993; Näslund et al., 2013), this is, to our knowledge, the first study using p-CPA to address the possible causal relationship between baseline differences in serotonergic transmission and EPM behavior. While it is tempting to regard the anxiety-reducing effect of a TPH2 inhibitor in animals displaying both enhanced anxiety and enhanced TPH2 expression as support for differences in serotonergic transmission to be of importance for the differences in anxiety, the possibility that the lack of an anxiety-reducing effect of p-CPA in HO rats reflects merely a ceiling effect should not be excluded. The fact that the anxiolytic effect of benzodiazepines in the same paradigm has never been reported to be confined to a specific subset of the tested animals however argues against this possibility; for example, in a study comparing Wistar rats selectively bred to display high (HAB) or low (LAB) anxiety-like behavior on the basis of their EPM performance, both strains displayed an anxiolytic-like response to a benzodiazepine, the difference between HAB and LAB remaining significant in treated animals (Liebsch et al., 1998).

It should also be underlined that the observed effects of p-CPA in LO and HO rats, respectively, refute the possibility that the apparent increase in serotonergic transmission in LO rats should be regarded as a compensatory mechanism aiming to dampen anxiety enhanced by other mechanisms; thus, had this been the case, p-CPA should enhance anxiety-like behavior in LO animals and amplify rather than counteract the difference between LO and HO animals. Likewise, had serotonin exerted an anxiety-dampening influence, administration of paroxetine should have enhanced rather than further reduced open arm activity in LO rats.

When interpreting the TPH2 elevation observed in the present study, it should be noted that LO rats displayed enhanced expression not only of TPH2 but also of other genes encoding proteins expressed by serotonergic neurons, such as the monoamine oxidase subtype expressed by serotonergic neurons (MAO-B), the serotonin transporter, aromatic L-amino acid decarboxylase, and a transcription factor of importance for the development of these neurons, fev. LO rats hence seem characterized either by a more developed serotonergic network or by serotonergic neurons displaying a more active transcription machinery compared with less anxious animals. The notion that LO rats are characterized by a stronger serotonergic innervation gains indirect support from a report by Keck and co-workers (2005) showing the above-mentioned HAB rats to display enhanced serotonin transporter binding in hippocampus and enhanced serotonin release in the same brain region when exposed to a stressor (in the form of an EPM session) in conjunction with serotonin reuptake inhibition. In the present study, serotonin levels were unfortunately not assessed using microdialysis but merely in brain homogenates, and the animals had not been exposed to a stressor shortly before sacrifice. It should be noted, however, that LO rats did display higher levels of serotonin in a brain region of critical importance for the regulation of anxiety, the amygdala; in contrast, no differences were observed in hippocampus or striatum. The lack of increase in the levels of the serotonin metabolite 5-HIAA in the amygdala of LO rats supports the notion that these animals, when in a restful situation, are not characterized by enhanced serotonin turnover.

Acute administration of an SRI to rodents causes elevated extracellular levels of serotonin in many (but not all) of the brain regions innervated by serotonergic nerve terminals (Rutter and Auerbach, 1993), which is well in line with the observation that this treatment, both in animals and humans, exerts a prompt dampening influence on functions that are normally under an inhibitory influence of serotonin, such as sexual behavior (McMahon, 2011) and anger/aggression (Landén et al., 2009). Previous studies (Griebel et al., 1994; Silva et al., 1999; Borsini et al., 2002; Drapier et al., 2007) suggesting acute administration of SRIs to exert an anxiogenic-like effect in the EPM are hence highly compatible with the notion that serotonin exerts an anxiety-enhancing effect in this paradigm. In the same vein, we also observed acute administration of an SRI, paroxetine, to exert an anxiety-enhancing effect, but only in the LO group. It is not farfetched to suggest that this difference between HO and LO rats with respect to the response to an indirect serotonin agonist is the result of LO rats displaying a more developed network of serotonergic nerve terminals and/or a larger capacity for serotonin formation; likewise, it may be speculated that the initial anxiogenic effect of SRIs in patients with anxiety disorders, such as panic disorder, which is seldom observed in nonanxious subjects, may be similarly explained. Notably, a study aiming to assess brain serotonin turnover by measuring jugular vein overflow of the serotonin metabolite 5-hydroxyindole acetic acid found support for a considerable increase in serotonin turnover in subcortical areas in patients with panic disorder (Esler et al., 2007).

It may seem unexpected that LO rats displayed *enhanced* expression of the serotonin transporter, given that anxiety-related traits in humans have been associated with the s allele of a polymorphism in the promoter of the serotonin transporter gene causing *reduced* expression of this protein (Sen et al., 2004). However, as discussed above, we suggest the enhanced expression of the serotonin transporter in LO rats to reflect an increase in the number of serotonergic neurons (or in the transcriptional activity of these) rather than an increase in the number of transporters per serotonergic neuron. While the net effect of the s allele on serotonergic output, taking also the possible effect during brain development into consideration, remains elusive, it is in fact not unlikely that this allele causes enhanced serotonergic output (as the result of impaired reuptake inhibition), which would be well in line with the results of this paper.

Likewise, it may seem counter-intuitive that proneness for anxiety should be associated with, and to some extent caused by, enhanced serotonergic output, given that SRIs, which are usually assumed to facilitate serotonergic transmission, are effective for most major anxiety disorders (Den Boer and Westenberg, 1988; Modigh et al., 1992; Nutt et al., 1999; Zohar and Westenberg, 2000). However, as discussed above, the anxiety-reducing effect of SRIs, unlike, for example, the effects of the same drugs on sexual functioning (McMahon et al. 2011), on certain forms of anger and irritability (for refs, see Eriksson, 1999), and on affective lability (for refs, see Landén et al., 2009), requires long-term administration. Theoretically, this discrepancy may be explained by differences in the regulation of those serotonergic neurons regulating anxiety on the one hand and those influencing sexual behavior and anger on the other, the former but not the latter requiring long-term SRI administration for an enhancement of serotonergic output to be at hand. However, given the anxiogenic effect of acute SRI administration observed both in humans and rodents, an alternative possibility, according to which the delayed onset of action might instead be explained in terms of an adaptive downregulation of the influence of anxiety-provoking serotonergic synapses (Salchner and Singewald, 2006; Graeff and Zangrossi, 2010, Lazary et al., 2011), appears at least as attractive.

Previous studies on the possible influence of long-term SRI administration on the EPM paradigm (in rats not characterized with respect to baseline anxiety) have failed to reveal consistent results, some but not all showing a modest anxiogenic-like effect (Borsini et al., 2002). In the present study, long-term administration of escitalopram by the per oral route neither enhanced nor reduced baseline EPM behavior in LO rats but blunted the anxiogenic-like response to acute administration of paroxetine in these animals, so that HO and LO rats did no longer differ with respect to how they responded to this provocation.

It hence seems as if subchronic administration of an SRI in animals displaying enhanced baseline anxiety-like behavior, as well as indices of enhanced serotonergic neurotransmission, does lead to a downregulation of the anxiogenic-like influence of serotonin as reflected by the enhanced anxiety-like behavior elicited by acute SRI administration. In line with this, Salchner and Singewald (2006) reported chronic treatment with fluoxetine to counteract the potentiating effect of acute fluoxetine administration on both escape behavior and fos expression induced by airjet provocation in Sprague-Dawley rats. Downregulation of postsynaptic 5HT2C receptors, or structures beyond these, is one possible underlying mechanism for such an adaptive response to long-term SRI administration (Bristow et al., 2000).

When evaluating the inability of subchronic escitalopram to reduce baseline anxiety in LO animals, it should be considered that all animals, when retested under the influence of escitalopram, had been exposed to the EPM at an earlier occasion (ie, when being categorized as LO and HO, respectively). Previous studies thus suggest that prior experience of the test may abolish the anxiolytic effect of drugs such as benzodizapines (File et al., 1990). Although we did take precautions to minimize the influence of this one-trial tolerance phenomenon by undertaking the 2 EPM tests in different rooms (Schneider et al., 2011) and in spite of the fact that several weeks elapsed between the 2 tests (which is also reported to counteract this effect), it cannot be excluded that this factor nevertheless precluded the chance of detecting an anxiolytic effect of subchronic escitalopram administration. However, it should be noted that p-CPA did exert an anxiolytic effect in LO animals in spite of the fact that they had previous experience of the paradigm.

While long-term escitalopram treatment thus reduced the anxiety elicited by acute paroxetine in LO rats but failed to alter baseline anxiety-like behavior in these animals, HO animals exposed to the same treatment displayed enhanced baseline anxiety-like behavior, hence eliminating also this difference between HO and LO rats. Tentatively, the dominating net effect of long-term administration of SRIs on the serotonergic synapses regulating the studied behavior in these animals is to exert a modest enhancement from a low starting point rather than to downregulate an excessive influence.

The aim of experiment I was not only to assess the possible association between EPM performance and brain serotonergic activity but also to explore to what extent HO and LO rats differ with respect to another behavior tentatively reflecting human anxiety, unconditioned acoustic startle. While a study on the above-mentioned HAB and LAB rats, bred on the basis of EPM performance, suggests rats with high anxiety, as assessed using EPM, to display lower unconditioned (as well as fear-sensitized) startle than LAB animals (Yilmazer-Hanke et al., 2004), we observed no difference in this regard between LO and HO rats, which is well in line with previous studies showing no correlation between EPM and startle (Yilmazer-Hanke et al., 2002; de Oliveira et al., 2011). Prompted by the considerable comorbidity between anxiety disorders and depression, we also exposed our animals to a paradigm claimed to reflect depressive-like behavior, that is, the forced-swim test. Again, however, no difference between LO and HO rats was found, a finding in line with an earlier study also assessing rats subdivided on the basis of EPM performance (Ho et al., 2002).

This study has certain limitations. First, while the EPM paradigm having bearing on human anxiety gains support from the fact that GABA A-receptor-activating anxiolytics reliably reduce anxiety-like behavior in this model (Pellow and File, 1986) while drugs known to be anxiogenic in man exert the opposite effect (Yeung et al., 2013), it should be acknowledged that the possible relationship between the EPM paradigm and the various forms of human anxiety, including panic anxiety (Graeff and Zangrossi, 2010), is far from clear-cut. Thus, other factors, such as impulsivity (Soubrié, 1986), could also be of importance for the studied behavior. It is, for example, not inconceivable that the reduction in time spent on open arms in LO rats upon subchronic administration of escitalopram may reflect reduced impulsivity rather than enhanced anxiety-like behavior (Thiébot et al., 1985; Cherek et al., 2002). Second, we did not address the possibility that different serotonergic pathways originating in different raphe nuclei may exert differential effects on the studied behavior (Donner et al., 2012; Paul and Lowry, 2013). Third, it cannot be excluded that the 2 behavioral stressors to which all animals in experiment I were exposed between the first EPM and sacrifice, that is, the acoustic startle test and the forced swim stress, may exert a lasting impact on brain serotonergic transmission, hence contributing to the observed association between EPM behavior and serotonergic activity. Fourth, we have, in this study, refrained from exploring the possible differences between HO and LO rats with respect to other neurotransmitters that have been attributed importance for inter-individual differences in anxiety and that may interact with serotonin in this regard, such as the endocannabinoids (Lazary et al., 2009).

The major novel conclusions of the present data are that: 1) inter-individual differences within a batch of Wistar rats can be utilized to study the influence of serotonin on anxiety-like behavior, 2) enhanced serotonergic activity is not only *associated* with enhanced anxiety-like behavior but also seems to be an important *causal factor* underlying inter-individual differences in this regard, 3) the anxiogenic effect of acute administration of an SRI in Wistar rats is more pronounced in animals with enhanced baseline anxiety-like behavior than in those with low anxiety (hence corresponding to the human situation), and 4) subchronic administration of an SRI blunts the anxiogenic effect of the acute administration of another SRI in animals with high anxiety-like behaviour at baseline. Our data support the view that proneness for anxiety in humans may also be partly caused by an enhanced anxiogenic influence of serotonin and that this is the reason why acute administration of an SRI may elicit anxiety in susceptible individuals while subchronic administration of the same agents often have the opposite effect.

## Interest Statement

None.

## References

[CIT0001] Alttoa A Kõiv K Hinsley T Brass A Harro J (2010) Differential gene expression in a rat model of depression based on persistent differences in exploratory activity. Eur Neuropsychopharmacol. 20:288–300. 19854624 10.1016/j.euroneuro.2009.09.005

[CIT0002] Andrade TG Zangrossi H Jr Graeff FG (2013) The median raphe nucleus in anxiety revisited. J Psychopharmacol 27:1107–1115. 23999409 10.1177/0269881113499208

[CIT0003] Araragi N Lesch KP (2013) Serotonin (5-HT) in the regulation of depression-related emotionality: insight from 5-HT transporter and tryptophan hydroxylase-2 knockout mouse models. Curr Drug Targets 14:549–570. 23547810 10.2174/1389450111314050005

[CIT0004] Bell C Forshall S Adrover M Nash J Hood S Argyropoulos S Rich A Nutt DJ (2002) Does 5-HT restrain panic? A tryptophan depletion study in panic disorder patients recovered on paroxetine. J Psychopharmacol 16:5–14. 11949771 10.1177/026988110201600116

[CIT0005] Bertoglio LJ Carobrez AP (2002) Anxiolytic effects of ethanol and phenobarbital are abolished in test-experienced rats submitted to the elevated plus maze. Pharmacol Biochem Behav 73:963–969. 12213543 10.1016/s0091-3057(02)00958-9

[CIT0006] Borsini F Podhorna J Marazziti D (2002) Do animal models of anxiety predict anxiolytic-like effects of antidepressants? Psychopharmacology (Berl) 163:121–141. 12202959 10.1007/s00213-002-1155-6

[CIT0007] Borta A Wohr M Schwarting RK (2006) Rat ultrasonic vocalization in aversively motivated situations and the role of individual differences in anxiety-related behavior. Behav Brain Res 166:271–280. 16213033 10.1016/j.bbr.2005.08.009

[CIT0008] Bristow LJ O’Connor D Watts R Duxon MS Hutson PH (2000) Evidence for accelerated desensitisation of 5-HT(2C) receptors following combined treatment with fluoxetine and the 5-HT(1A) receptor antagonist, WAY 100,635, in the rat. Neuropharmacology 39:1222–1236. 10760364 10.1016/s0028-3908(99)00191-4

[CIT0009] Chamas F Serova L Sabban EL (1999) Tryptophan hydroxylase mRNA levels are elevated by repeated immobilization stress in rat raphe nuclei but not in pineal gland. Neurosci Lett 267:157–160. 10381000 10.1016/s0304-3940(99)00340-7

[CIT0010] Cherek DR Lane SD Pietras CJ Steinberg JL (2002) Effects of chronic paroxetine administration on measures of aggressive and impulsive responses of adult males with a history of conduct disorder. Psychopharmacology (Berl) 159:266–274. 11862359 10.1007/s002130100915

[CIT0011] de Oliveira LC Gomes MZ Brandão ML (2011) Influence of age on reactivity to diverse emotional challenges in low- and high-anxiety rats. Int J Dev Neurosci 29:77–83. 20833243 10.1016/j.ijdevneu.2010.08.006

[CIT0012] Den Boer JA Westenberg HG (1988) Effect of a serotonin and noradrenaline uptake inhibitor in panic disorder; a double-blind comparative study with fluvoxamine and maprotiline. Int Clin Psychopharmacol 3:59–74. 2833543 10.1097/00004850-198801000-00005

[CIT0013] Donner NC Johnson PL Fitz SD Kellen KE Shekhar A Lowry Ca (2012) Elevated tph2 mRNA expression in a rat model of chronic anxiety. Depress Anxiety 29:307–319. 22511363 10.1002/da.21925 PMC4414333

[CIT0014] Drapier D Bentué-Ferrer D Laviolle B Millet B Allain H Bourin M Reymann J-M (2007) Effects of acute fluoxetine, paroxetine and desipramine on rats tested on the elevated plus-maze. Behav Brain Res 176:202–209. 17095104 10.1016/j.bbr.2006.10.002

[CIT0015] Eison MS (1990) Serotonin: a common neurobiologic substrate in anxiety and depression. J Clin Psychopharmacol 10:26S–30S. 2198299

[CIT0016] El Khoury A Gruber SH Mørk A Mathé AA (2006) Adult life behavioral consequences of early maternal separation are alleviated by escitalopram treatment in a rat model of depression. Prog Neuropsychopharmacol Bio Psychiatry 20:535–540. 16414167 10.1016/j.pnpbp.2005.11.011

[CIT0017] Eriksson E (1999) Serotonin reuptake inhibitors for the treatment of premenstrual dysphoria. Int Clin Psychopharmacol 14:S27–33. 10471170

[CIT0018] Esler M Lambert E Alvarenga M Socratous F Richards J Barton D Pier C Brenchley C Dawood T Hastings J Guo L Haikerwal D Kaye D Jennings G Kalff V Kelly M Wiesner G Lambert G (2007) Increased brain serotonin turnover in panic disorder patients in the absence of a panic attack: reduction by a selective serotonin reuptake inhibitor. Stress 10:295–304. 17613943 10.1080/10253890701300904

[CIT0019] Fernandez SP Gaspar P (2012) Investigating anxiety and depressive-like phenotypes in genetic mouse models of serotonin depletion. Neuropharmacology 62:144–154. 21945798 10.1016/j.neuropharm.2011.08.049

[CIT0020] File SE Mabbutt PS Hitchcott PK (1990) Characterisation of the phenomenon of “one-trial tolerance” to the anxiolytic effect of chlordiazepoxide in the the elevated plus-maze. Psychopharmacology (Berl) 102:98–101 1975449 10.1007/BF02245751

[CIT0021] Furlan PM Kallan MJ Have TT Lucki I Katz I (2004) SSRIs do not cause affective blunting in healthy elderly volunteers. Am J Geriatr Psychiatry 12:323–30. 15126234

[CIT0022] Furmark T Henningsson S Appel L Ahs F Linnman C Pissiota A Faria V Oreland L Bani M Pich EM Eriksson E Fredrikson M (2009) Genotype over-diagnosis in amygdala responsiveness: affective processing in social anxiety disorder. J Psychiatry Neurosci 34:30–40. 19125211 PMC2612081

[CIT0023] Gardner KL Hale MW Oldfield S Lightman SL Plotsky PM Lowry CA (2009) Adverse experience during early life and adulthood interact to elevate tph2 mRNA expression in serotonergic neurons within the dorsal raphe nucleus. Neuroscience 163:991–1001. 19647049 10.1016/j.neuroscience.2009.07.055 PMC2760611

[CIT0024] Graeff FG Zangrossi H (2010) The dual role of serotonin in defense and the mode of action of antidepressants on generalized anxiety and panic disorders. Cent Nerv Syst Agents Med Chem 10:207–217. 20528764 10.2174/1871524911006030207

[CIT0025] Griebel G Moreau J-L Jenck F Misslin R Martin JR (1994) Acute and chronic treatment with 5-HT reuptake inhibitors differentially modulate emotional responses in anxiety models in rodents. Psychopharmacology 113:463–470. 7862860 10.1007/BF02245224

[CIT0026] Hariri AR Mattay VS Tessitore A Kolachana B Fera F Goldman D Egan MF Weinberger DR (2002) Serotonin transporter genetic variation and the response of the human amygdala. Science 297:400–403. 12130784 10.1126/science.1071829

[CIT0027] Ho Y-J Eichendorff J Schwarting RKW (2002) Individual response profiles of male Wistar rats in animal models for anxiety and depression. Behav Brain Res 136:1–12. 12385785 10.1016/s0166-4328(02)00089-x

[CIT0028] Jahanshahi A Le Maitre E Temel Y Lanfumey L Hamon M Lesch KP Tordera RM Del Rio J Aso E Maldonado R Hokfelt T Steinbusch HW (2011) Altered expression of neuronal tryptophan hydroxylase-2 mRNA in the dorsal and median raphe nuclei of three genetically modified mouse models relevant to depression and anxiety. J Chem Neuroanat 41:227–233. 21704153 10.1016/j.jchemneu.2011.05.015

[CIT0029] Keck ME Sartori SB Welt T Müller MB Ohl F Holsboer F Landgraf R Singewald N (2005) Differences in serotonergic neurotransmission between rats displaying high or low anxiety/depression-like behaviour: effects of chronic paroxetine treatment. J Neurochem 92:1170–1179. 15715667 10.1111/j.1471-4159.2004.02953.x

[CIT0030] Landén M Erlandsson H Bengtsson F Andersch B Eriksson E (2009) Short onset of action of a serotonin reuptake inhibitor when used to reduce premenstrual irritability. Neuropsychopharmacology 34:585–592. 18596686 10.1038/npp.2008.86

[CIT0031] Lazary J Lazary A Gonda X Benko A Molnar E Hunyady L Juhasz G Bagdy G (2009) Promoter variants of the cannabinoid receptor 1 gene (CNR1) in interaction with 5-HTTLPR affect the anxious phenotype. Am J Med Genet B Neuropsychiatr Genet 150:1118–1127 19725030 10.1002/ajmg.b.31024

[CIT0032] Lazary J Juhasz G Hunyady L Bagdy G (2011) Personalized medicine can pave the way for the safe use of CB1 receptor antagonists. Trends Pharmacol Sci 32:270–280 21497918 10.1016/j.tips.2011.02.013

[CIT0033] Lesch KP Bengel D Heils A Sabol SZ Greenberg BD Petri S Benjamin J Muller CR Hamer DH Murphy DL (1996) Association of anxiety-related traits with a polymorphism in the serotonin transporter gene regulatory region. Science 274:1527–1531. 8929413 10.1126/science.274.5292.1527

[CIT0034] Liebsch G Linthorst AC Neumann ID Reul JM Holsboer F Landgraf R (1998) Behavioral, physiological, and neuroendocrine stress responses and differential sensitivity to diazepam in two Wistar rat lines selectively bred for high- and low–anxiety-related behavior. Neuropsychopharmacology 19:381–396. 9778660 10.1016/S0893-133X(98)00042-6

[CIT0035] Maron E Nutt D Shlik J (2012) Neuroimaging of serotonin system in anxiety disorders. Curr Pharm Des 18:5699–5708. 22632475 10.2174/138161212803530844

[CIT0036] McMahon CG (2011) Efficacy of dapoxetine in the treatment of premature ejaculation. Clin Med Insights Reprod Health 5:25–39. 24453509 10.4137/CMRH.S7337 PMC3888071

[CIT0037] Melke J Westberg L Nilsson S Landén M Söderström H Baghaei F Rosmond R Holm G Bjorntorp P Nilsson LG Adolfsson R Eriksson E (2003) A polymorphism in the serotonin receptor 3A (HTR3A) gene and its association with harm avoidance in women. Arch Gen Psychiatry 60:1017–1023. 14557147 10.1001/archpsyc.60.10.1017

[CIT0038] Modigh K Westberg P Eriksson E (1992) Superiority of clomipramine over imipramine in the treatment of panic disorder: a placebo-controlled trial. J Clin Psychopharmacol 12:251–261. 1527228

[CIT0039] Mosienko V Bert B Beis D Matthes S Fink H Bader M Alenina N (2012) Exaggerated aggression and decreased anxiety in mice deficient in brain serotonin. Transl Psychiatry 2:e122. 22832966 10.1038/tp.2012.44 PMC3365263

[CIT0040] Näslund J Studer E Nilsson K Westberg L Eriksson E (2013) Serotonin depletion counteracts sex differences in anxiety-related behaviour in rat. Psychopharmacology (Berl) 230:29–35. 23681161 10.1007/s00213-013-3133-6

[CIT0041] Nutt DJ Forshall S Bell C Rich A Sandford J Nash J Argyropoulos S (1999) Mechanisms of action of selective serotonin reuptake inhibitors in the treatment of psychiatric disorders. Eur Neuropsychopharmacol 9:S81–86. 10523062 10.1016/s0924-977x(99)00030-9

[CIT0042] Paul ED Lowry CA (2013) Functional topography of serotonergic systems supports the Deakin/Graeff hypothesis of anxiety and affective disorders. J Psychopharmacol 27:1090–1106. 23704363 10.1177/0269881113490328

[CIT0043] Pellow S File SE (1986) Anxiolytic and anxiogenic drug effects on exploratory activity in an elevated plus-maze: a novel test of anxiety in the rat. Pharmacol Biochem Behav 24:525–529. 2871560 10.1016/0091-3057(86)90552-6

[CIT0044] Pinheiro SH Zangrossi H Jr Del-Ben CM Graeff FG (2007) Elevated mazes as animal models of anxiety: effects of serotonergic agents. An Acad Bras Cienc 79:71–85. 17401477 10.1590/s0001-37652007000100010

[CIT0045] Rammsayer T Netter P (1990) Personality related differences in response to 5-HT uptake inhibition. Int J Neurosci 55:99–106. 2128079 10.3109/00207459008985955

[CIT0046] Ramos RT Gentil V Gorenstein C (1993) Clomipramine and initial worsening in panic disorder: beyond the ‘jitteriness syndrome’. J Psychopharmacol (Oxford, England) 7:265–269. 10.1177/026988119300700305 22290840

[CIT0047] Rutter JJ Auerbach SB (1993) Acute uptake inhibition increases extracellular serotonin in the rat forebrain. J Pharmacol Exp Ther 265:1319–1324. 7685386

[CIT0048] Salchner P Singewald N (2006) 5-HT receptor subtypes involved in the anxiogenic-like action and associated Fos response of acute fluoxetine treatment in rats. Psychopharmacology (Berl) 185:282–288. 16521035 10.1007/s00213-005-0247-5

[CIT0049] Schneider P Ho Y-J Spanagel R Pawlak CR (2011) A novel elevated plus-maze procedure to avoid the one-trial tolerance problem. Front Behav Neurosci 5:43–43. 21845176 10.3389/fnbeh.2011.00043 PMC3146044

[CIT0050] Schwarting RK Thiel CM Müller CP Huston JP (1998) Relationship between anxiety and serotonin in the ventral striatum. Neuroreport 9:1025–1029. 9601661 10.1097/00001756-199804200-00013

[CIT0051] Sen S Burmeister M Ghosh D (2004) Meta-analysis of the association between a serotonin transporter promoter polymorphism (5-HTTLPR) and anxiety-related personality traits. Am J Med Genet B Neuropsychiatr Genet 127B:85–89. 15108187 10.1002/ajmg.b.20158

[CIT0052] Sidor MM Amath A MacQueen G Foster JA (2010) A developmental characterization of mesolimbocortical serotonergic gene expression changes following early immune challenge. Neuroscience 171:734–746. 20816924 10.1016/j.neuroscience.2010.08.060

[CIT0053] Silva MT Alves CR Santarem EM (1999) Anxiogenic-like effect of acute and chronic fluoxetine on rats tested on the elevated plus-maze. Braz J Med Biol Res 32:333–339. 10347793 10.1590/s0100-879x1999000300014

[CIT0054] Simmons JG Allen NB (2011) Mood and personality effects in healthy participants after chronic administration of sertraline. J Affect Disord 134:377–385. 21724265 10.1016/j.jad.2011.06.007

[CIT0055] Sinclair LI Christmas DM Hood SD Potokar JP Robertson A Isaac A Srivastava S Nutt DJ Davies SJ (2009) Antidepressant-induced jitteriness/anxiety syndrome: systematic review. Br J Psychiatry 194:483–490. 19478285 10.1192/bjp.bp.107.048371

[CIT0056] Soubrié P (1986) Reconciling the role of central serotonin neurons in human and animal behavior. Behav Brain Sci 9:319–319.

[CIT0057] Targum SD Marshall LE (1989) Fenfluramine provocation of anxiety in patients with panic disorder. Psychiatry Res 28:295–306. 2669003 10.1016/0165-1781(89)90210-2

[CIT0058] Thiébot MH Le Bihan C Soubrié P Simon P (1985) Benzodiazepines reduce the tolerance to reward delay in rats. Psychopharmacology (Berl) 86:147–152. 2862657 10.1007/BF00431700

[CIT0059] Treit D Robinson A Rotzinger S Pesold C (1993) Anxiolytic effects of serotonergic interventions in the shock-probe burying test and the elevated plus-maze test. Behav Brain Res 54:23–34. 8504010 10.1016/0166-4328(93)90045-r

[CIT0060] Yeung M Lu L Hughes AM Treit D Dickson CT (2013) FG7142, yohimbine, and betaCCE produce anxiogenic-like effects in the elevated plus-maze but do not affect brainstem activated hippocampal theta. Neuropharmacology 75:47–52. 23851259 10.1016/j.neuropharm.2013.06.027

[CIT0061] Yilmazer-Hanke DM Faber-Zuschratter H Linke R Schwegler H (2002) Contribution of amygdala neurons containing peptides and calcium-binding proteins to fear-potentiated startle and exploration-related anxiety in inbred Roman high- and low-avoidance rats. Eur J Neurosci 15:1206–1218. 11982631 10.1046/j.1460-9568.2002.01945.x

[CIT0062] Yilmazer-Hanke DM Wigger a Linke R Landgraf R Schwegler H (2004) Two Wistar rat lines selectively bred for anxiety-related behavior show opposite reactions in elevated plus maze and fear-sensitized acoustic startle tests. Behav Genet 34:309–318. 14990869 10.1023/B:BEGE.0000017874.40934.64

[CIT0063] Zohar J Westenberg HGM (2000) Anxiety disorders: a review of tricyclic antidepressants and selective serotonin reuptake inhibitors. Acta Psychiatr Scand 101:39–49. 10.1111/j.1600-0447.2000.tb10947.x 11019934

